# PCSK9 plasma concentration is associated with epicardial adipose tissue volume and metabolic control in patients with type 1 diabetes

**DOI:** 10.1038/s41598-024-57708-5

**Published:** 2024-03-26

**Authors:** Helena Sardà, Cristina Colom, Sonia Benitez, Gemma Carreras, Judit Amigó, Inka Miñambres, David Viladés, Francisco Blanco-Vaca, Jose Luís Sanchez-Quesada, Antonio Pérez

**Affiliations:** 1 Department of Endocrinology and Nutrition, Hospital de la Santa Creu i Sant Pau - Hospital Dos de Maig, Antoni Maria Claret, 167, 08025 Barcelona, Spain; 2 Department of Medicine, Universitat Autònoma de Barcelona, Bellaterra, Spain; 3 Cardiovascular Biochemistry Group, Institut de Recerca Sant Pau (IR Sant Pau), Sant Quintí, 77-79, 08041 Barcelona, Spain; 4 CIBER en Diabetes y Enfermedades Metabólicas (CIBERDEM), Madrid, Spain; 5 Department of Pediatrics, Hospital de la Santa Creu i Sant Pau, Barcelona, Spain; 6 Department of Pediatrics, Obstetrics and Gynecology, and Preventive Medicine and Public Health, Universitat Autònoma de Barcelona, Bellaterra, Spain; 7 Department of Endocrinology and Nutrition, Hospital Universitari Vall d’Hebrón, Barcelona, Spain; 8 Cardiac Imaging Unit, Cardiology Department, Hospital de la Santa Creu i Sant Pau, Barcelona, Spain; 9 Centro de Investigación en red de enfermedades cardiovasculares (CIBERCV), Madrid, Spain; 10 Department of Clinical Biochemistry, Hospital de la Santa Creu i Sant Pau, IIB Sant Pau, Barcelona, Spain; 11 Department of Biochemistry and Molecular Biology, Universitat Autònoma de Barcelona, Bellaterra, Spain

**Keywords:** Type 1 diabetes, Biomarkers, Epicardial adipose tissue, Cardiometabolic risk factors, Cardiometabolic traits, Cardiovascular disease risk, Endocrine system and metabolic diseases, Endocrinology, Medical research, Biomarkers, Predictive markers

## Abstract

Patients with type 1 diabetes (T1D) have a greater risk of cardiovascular disease. Proconvertase subtilisin-kexin 9 (PCSK9) is involved in the atherosclerosis process. This study aimed to determine the relationship between PCSK9 levels and epicardial adipose tissue (EAT) volume and cardiometabolic variables in patients with T1D. This was an observational cross-sectional study including 73 patients with T1D. Clinical, biochemical and imaging data were collected. We divided the patients into two groups according to their glycemic control and the EAT index (iEAT) percentile. We performed a correlation analysis between the collected variables and PCSK9 levels; subsequently, we performed a multiple regression analysis with the significant parameters. The mean age was 47.6 ± 8.5 years, 58.9% were men, and the BMI was 26.9 ± 4.6 kg/m^2^. A total of 31.5%, 49.3% and 34.2% of patients had hypertension, dyslipidemia and smoking habit, respectively. The PCSK9 concentration was 0.37 ± 0.12 mg/L, which was greater in patients with worse glycemic control (HbA1c > 7.5%), dyslipidemia and high EAT volume (iEAT > 75th percentile). The PCSK9 concentration was positively correlated with age (r = 0.259; p = 0.027), HbA1c (r = 0.300; p = 0.011), insulin dose (r = 0.275; p = 0.020), VLDL-C level (r = 0.331; p = 0.004), TG level (r = 0.328; p = 0.005), and iEAT (r = 0.438; p < 0.001). Multiple regression analysis revealed that 25% of the PCSK9 variability was explained by iEAT and HbA1c (p < 0.05). The PCSK9 concentration is associated with metabolic syndrome parameters, poor glycemic control and increased EAT volume in patients with T1D.

## Introduction

Patients with type 1 diabetes (T1D) have a greater risk of cardiovascular disease (CVD)^1–3^ than does the general population. The molecular processes underlying this risk are unclear, and multiple mechanisms have been proposed. Hyperglycemia and hypoglycemia contribute to mechanisms related to atherosclerosis, such as oxidative stress, nonenzymatic glycosylation, endothelial dysfunction and inflammatory pathways^3–5^. The presence of diabetic kidney disease (DKD), metabolic syndrome, insulin resistance and obesity in patients with T1D dramatically increases the risk of CVD^3,4,6^. Although plasma cholesterol and triglyceride levels are usually normal in patients with T1D, lipoprotein composition may be altered, with dysfunctional and proatherogenic HDL lipoproteins^6^, which could partly account for the increased cardiovascular risk. However, these risk factors and comorbidities do not fully explain the heightened risk observed, suggesting that other factors could be involved.

Epicardial adipose tissue (EAT) is a visceral fat depot surrounding the myocardium and coronary arteries under the visceral pericardium. It is an important source of nutrients for cardiomyocytes and favors homeostasis of the myocardium, but in some conditions, it can also produce a wide range of inflammatory mediators^7,8^. Several studies have demonstrated that increased EAT volume is a cardiovascular risk factor^9–11^, and Shmilovich et al.^13^ postulated that the threshold value of the EAT index (iEAT) that predicts the development of major cardiovascular events in a cohort of healthy individuals was the 95th percentile, which corresponded to 68.1 cm^3^/m^2^ of body surface area. In type 2 diabetes or obesity, there is an increase in the volume of epicardial adipose tissue, which implies an increased risk of developing CVD^8,12^.

On the other hand, increased levels of the circulating proconvertase subtilisin-kexin 9 (PCSK9) are found in patients with type 2 diabetes, obesity and CVD and are associated with CVD severity^14–18^. The main action of PCSK9 is the regulation of low-density lipoprotein receptor (LDLR) recycling, forming a complex that is internalized and promotes the degradation of LDLR by hepatocytes. LDLR function is to bind plasmatic low-density lipoprotein (LDL) and remove it from circulation to supply cholesterol to peripheral cells. However, in addition to its role in lipid metabolism, PCSK9 is involved in various processes related to atherosclerosis, affecting both endothelial cell and cardiomyocyte function and proinflammatory pathways^19–21^.

As T1D is increasingly associated with insulin resistance and obesity the investigation of these two parameters, EAT volume and PCSK9 concentration, in this setting, could add new information in this population. This study aimed to determine the relationship between circulating PCSK9 levels, metabolic status and EAT volume in patients with T1D.

## Methods

This was an observational transversal study including 73 Caucasian patients with T1D who were followed since their diagnosis between 1985 and 1994 at a tertiary university hospital in Barcelona, ​​Spain^22^. The diagnosis of T1D was established according to international guidelines^23^. All the subjects provided written informed consent to participate. The protocol was approved by the clinical research ethics committee of the Hospital de la Santa Creu i Sant Pau (IIBSP-DIA-2011-114). All procedures performed involving human participants were in accordance with the 1964 Helsinki declaration and its later amendments or comparable ethical standards.

The following baseline characteristics were recorded: sex, age, body mass index (BMI), waist circumference (WC), time since diagnosis, type of therapy (basal-bolus regimen or use of continuous subcutaneous insulin infusion), dose of insulin expressed as units per kg per day (UI/kg/day), smoking status and the presence of comorbidities/chronic diabetic complications. Dyslipidemia was defined as the presence of any of the following: triglyceride (TG) ≥ 1.7 mmol/L, LDL cholesterol (LDL-C) > 4.2 mmol/L or hypolipidemic treatment. Hypertension was defined as the presence of three or more systolic blood pressure measurements ≥ 140 mmHg and/or diastolic blood pressure measurements ≥ 90 mmHg or antihypertensive treatment. Overweight was defined as a BMI ranging from 25 to 29.9 kg/m^2^, and obesity was considered to be present if the BMI was greater than or equal to 30 kg/m^2^. High WC was defined as a WC > 102 cm in men and > 88 cm in women. A hypertriglyceridemic waist was defined as a high WC and TG ≥ 1.7 mmol/L.

The biochemical parameters analyzed included total cholesterol (TC), TG, high-density lipoprotein cholesterol (HDL-C), LDL-C, very low-density lipoprotein cholesterol (VLDL-C), apolipoprotein B (ApoB), HbA1c levels and the urine albumin-to-creatinine ratio (ACR), as previously described^22^. The PCSK9 concentration was determined via ELISA according to the manufacturer’s instructions (Bio Vendor, Ref # RD191473200R).

A cardiac computed tomography angiography (CCTA) exam using a 256-slice CT scanner (Brilliance iCT 256; Philips Healthcare, Amsterdam, the Netherlands) was performed on all participants. The data is expressed as the EAT volume (cm^3^) indexed to the body surface area (m^2^), EAT index cm^3^/m^2^. The details of the procedure are described in detail in^22,24^.

The statistical analyses were performed using the Statistical Package for the Social Sciences (SPSS, Inc., Chicago, IL, USA) version 21.0 for Windows. Variables are expressed as the mean ± standard deviation (SD) for continuous variables and as absolute numbers with percentages for categorical variables. The Kolmogorov‒Smirnov test was used to assess the distribution of continuous variables. We used the parametric chi-square test to compare categorical variables. Student’s t test was used to compare categorical and continuous variables with a normal distribution, and the Mann–Whitney U test was performed for nonparametric variables. The Pearson correlation test was used to analyze the correlation between continuous variables with a normal distribution, and Spearman’s rho test was performed for the nonparametric variables. Statistically significant correlations between the studied variables and the PCSK9 concentration were further analyzed by a multiple regression model and the backward method, in which nonparametric variables were previously transformed into logarithms. p < 0.05 was considered to indicate statistical significance.

### Ethics approval and consent to participate

The protocol was approved by the clinical research ethics committee of the Hospital de la Santa Creu i Sant Pau (IIBSP-DIA-2011-114). All the subjects provided written informed consent to participate.

## Results

The baseline demographic, clinical and biochemical characteristics of the 73 patients included are shown in Table 1. There were 15 (20.5%) patients with obesity, 43 (58.9%) with overweight, 20 (27.4%) with high WC, and 5 (6.8%) with hypertriglyceridemic waist. The mean iEAT was 39.8 ± 22.4 cm^3^/m^2^ and 16 subjects (21.9%) had an iEAT > 75th percentile.

**Table 1:** Baseline clinical, metabolic and biochemical characteristics.

Sex M/F	43 (58.9)/30 (41.1)
Age, years	47.2 ± 8.5
Time since diagnose, years	22.4 ± 2.2
Insulin dose, Ui/kg/day	0.6 ± 0.2
Weight, kg	77.8 ± 17.7
BMI, kg/m^2^	26.9 ± 4.6
WC, cm	93.9 ± 13.1
Type of treatment, BBT/CSII	62 (84.9)/11 (15.1)
Smoking status	
Smoker	25 (34.2)
Former smoker	22 (30.1)
Non smoker	26 (35.6)
Dyslipidemia	40 (54.8)
Hypolipidemic treatment	34 (46.6)
Statin treatment	33 (45.2)
Statins and ezetimibe treatment	1 (1.4)
Hypertension	23 (31.5)
Diabetic retinopathy	13 (18.1)
Diabetic nephropathy	8 (11)
Diabetic neuropathy	12 (16.4)
CAD	1 (1.4)
Stroke	1 (1.4)
Peripheral arterial disease	2 (2.8)
HbA1c, % (mmol/mol)	7.6 (60 mmol/mol) ± 1.1
ACR, mg/mmol	2.2 ± 0.7
Total cholesterol, mmol/L	4.77 ± 0.72
HDL cholesterol, mmol/L	1.48 ± 0.30
LDL cholesterol, mmol/L	2.83 ± 0.56
VLDL cholesterol, mmol/L	0.45 ± 0.32
Triglycerides, mmol/L	1.02 ± 0.9
Apolipoprotein B, g/L	0.78 ± 0.16
PCSK9, mg/L,	0.37 ± 0.12

Male (M); Female (F); Waist circumference (WC); Basal-bolus therapy (BBT); Continuous Subcutaneous Insulin Infusion (CSII); Coronary artery disease (CAD); Urine Albumin to Creatinine Ratio (ACR); Proconvertase subtilisin-kexin 9 (PCSK9).

The data are expressed as the mean ± SD or n (%).

**Figure 1:**
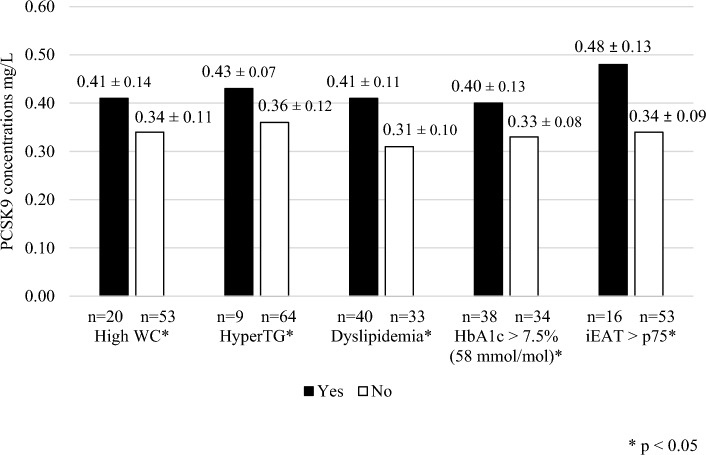
Comparative PCSK9 plasma concentration according to clinical and biochemical variables. Figure shows the difference in the PCSK9 concentration according to clinical, metabolic and biochemical parameters. Proconvertase subtilisin-kexin 9 (PCSK9); Waist circumference (WC); Hypertriglyceridemia (HyperTG); Epicardial adipose tissue index (iEAT).

The mean PCSK9 concentration was 0.37 ± 0.12 mg/L. The PCSK9 concentration was significantly greater in T1D patients with a high WC, TG ≥ 1.7 mmol/L, dyslipidemia, HbA1c > 7.5% (58 mmol/mol), or an iEAT above the 75th percentile (Fig. 1). We did not find differences in the PCSK9 plasma concentration according to sex or with hypertension, obesity, overweight, chronic complications, or smoking (data not shown).

**Figure 2:**
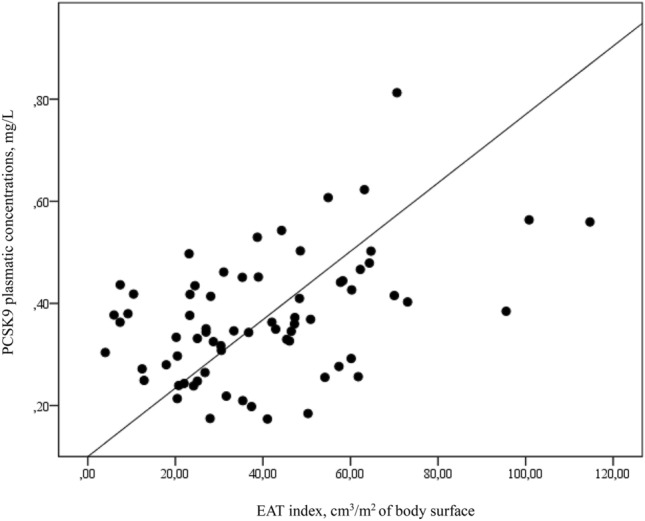
Correlation between EAT index and PCSK9 plasmatic concentration. Figure shows the correlation between the PCSK9 plasma concentration and the EAT index. r = 0.438, p < 0.001. Proconvertase subtilisin-kexin 9 (PCSK9); Epicardial adipose tissue (EAT).

Correlation analysis revealed positive correlations between PCSK9 concentration and age (r = 0.259; p = 0.027), HbA1c (r = 0.300; p = 0.011), insulin dose (r = 0.275; p = 0.020), VLDL-C level (r = 0.331; p = 0.004), TG level (r = 0.328; p = 0.005), and iEAT (r = 0.438; p < 0.001). The correlation between PCSK9 and iEAT is shown in Fig. 2.

Among the quantitative variables significantly correlated with the PCSK9 plasma concentration, multiple regression analysis was performed to analyze the predictors of the PCSK9 concentration; these included age, HbA1c, insulin dose, TG level and iEAT. The results showed that 25% of the PCSK9 variability was explained by iEAT and HbA1c (p < 0.05) (Table 2).

**Table 2:** Multiple linear regression function for PCSK9.

	Nonstandardized coefficients		Typified coefficients	t	P value	IC (95%) for B
	B	Typical error				
iEAT	0.002	0.002	0.374	3.389	0.001	0.001 to 0.003
HbA1c	0.029	0.012	0.266	2.411	0.019	0.005 to 0.052
Constant	0.070	0.091		0.769	0.445	− 0.112 to 0.252

To evaluate the adjustment of the regression model to the observed data, different statistics were used, such as the multiple correlation coefficient (R = 0.498) and the coefficient of determination (R2 = 0.248). The R2 value indicates that 25% of the variation in PCSK9 is explained by the model. The corrected R2 is 0.225. The significance value of F (Fisher distribution) = 10.56 with a P value < 0.001 confirms that the model is appropriate.

Proconvertase subtilisin-kexin 9 (PCSK9); Epicardial adipose tissue index (iEAT).

## Discussion

**Figure 3:**
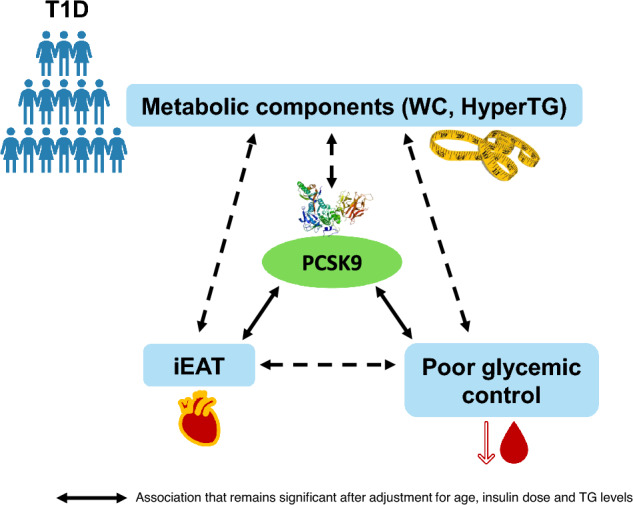
A graphical summary of results is presented in this figure. Elevated plasma concentration of PCSK9 is associated with components of metabolic syndrome, poor glycemic control and high EAT volume in patients with T1D. Type 1 diabetes (T1D); Waist circumference (WC); Hypertriglyceridemia (HyperTG); Proconvertase subtilisin-kexin 9 (PCSK9); Epicardial adipose tissue index (iEAT).

In the present study, we confirm the relationship between the PCSK9 concentration and the degree of metabolic control in patients with T1D and also with components of metabolic syndrome. The main novelty of our study is the positive association between the iEAT and the PCSK9 plasma concentration, which remained significant after adjustment for other confounding variables, such as age, insulin dose and TG level (Fig. 3).

### PCSK9 and glycemic control

A higher concentration of PCSK9 has been described in T1D patients with poor glycemic control^25–27^. Some studies have shown that insulin induces PCSK9 expression, principally through SREBP-1c and HNF-1α transcription factors^28–31^, suggesting a potential link between poor glycemic control, insulin dose and PCSK9 levels in patients with T1D. However, Laugier-Robiolle et al.^25^ reported that the association between PCSK9 concentration and poor glycemic control remained significant after adjusting for insulin dose, as occurred in our cohort. In vitro studies have demonstrated that the increase in cholesterol concentration, may lead to β-cell dysfunction and worsening/progression of diabetes^32,33^. In this context, we could hypothesize that the association between PCSK9 levels and glycemic control is mediated by increased cholesterol concentration. However, in subjects with familial hypercholesterolemia, including those with a gain of function mutation in PCSK9, epidemiological studies have shown a lower DM prevalence^34^.Thus, the underlying mechanism explaining the relationship between PCSK9 concentration and poor glycemic control remains to be clarified.

### PCSK9 and components of metabolic syndrome

The relationship between PCSK9 levels and insulin resistance parameters has been reported in previous studies in patients with and without type 2 diabetes^35–37^, but information on T1D is scarce. In general population and in patients with type 2 diabetes several studies have reported an association of PCSK9 levels with obesity^38–40^, WC and different metabolic parameters^35^such as TG and HOMA score^35^. In our study we also found a relationship between increased levels of PCSK9 in patients with high WC, but not with overweight and obesity. Concerning atherogenic dyslipidemia, the association between PCSK9 concentration and elevated TG levels or the prevalence of small dense LDL (sdLDL) has already been described in previous studies^25,27,41,42^ in patients with T1D and type 2 diabetes^25,27,41,42^. In our cohort, we also found an association between increased levels of PCSK9 and the presence of dyslipidemia and hyperTG. Notably, treatment with statins and fibrates increases plasma PCSK9 levels^19,41,43^, likely due to counterregulation of LDLR overexpression caused by statins^43^. Although this could partially explain the association between the PCSK9 concentration and dyslipidemia found in our study, this fact does not justify the association with the presence of hyperTG because none of the patients were receiving treatment with fibrates.

### PCSK9 and iEAT

Studies concerning the relationship between PCSK9 and EAT are scarce, and no study has analyzed such association in patients with T1D. Dozio et al. studied the relationship between PCSK9 and EAT in subjects with CVD. They found a positive correlation between the local expression of PCSK9 by EAT and its thickness but they did not find a positive association between plasma PCSK9 levels and EAT volume in these patients^44^. The authors argue that the EAT-induced inflammation may upregulate PCSK9 expression, thus explaining the association between the local expression of PCSK9 by EAT and its thickness^44^. In contrast, Baragetti et al.^45^ reported that nondiabetic individuals with the loss-of-function PCSK9 R46L variant, which implies decreased PCSK9 concentration, had greater epicardial fat thickness. These discrepancies point to a complex relationship between PCSK9 alterations and the accumulation of EAT, which deserves future research. As EAT thickness is a marker for visceral adiposity and the related metabolic and cardiovascular risk, PCSK9 might be one of the factors responsible for the development of atherogenic dyslipidemia and increased CVD risk in patients with T1D.The main limitations of our study are its cross-sectional nature and the small sample size of our cohort, making larger studies necessary to confirm our data. Another limitation of the study is the presence of statin treatment, which could partially explain the association between the PCK9 concentration and dyslipidemia, although would not justify the association with hyperTG because none of the patients were receiving treatment with fibrates. However, the main strength of this study is that, to the best of our knowledge, this is the first study to evaluate parameters related to metabolic syndrome and EAT measured by CCTA in patients with T1D. Furthermore, this procedure has been performed in well-characterized patients with T1D, treated with intensive insulin treatment and followed in our hospital since their diagnosis.

## Conclusion

In patients with T1D, an elevated plasma concentration of PCSK9 is associated with components of metabolic syndrome, poor glycemic control and high EAT volume. EAT thickness is a marker for visceral adiposity and related metabolic alterations, and PCSK9 might be one of the factors responsible for the development of atherogenic dyslipidemia. In this context, further studies, including those with newly drugs such as monoclonal antibody against PCSK9 and synthetic small RNA that interferes PCSK9 transcription^46,47^, could contribute to acquire a more detailed understanding of the link between PCSK9 and EAT volume and to determine its contribution to dyslipidemia and increased CVD risk associated with T1D.

## Data Availability

The datasets used and/or analyzed during the current study are available from the corresponding author on reasonable request.
